# The PhyR homolog RSP_1274 of *Rhodobacter sphaeroides* is involved in defense of membrane stress and has a moderate effect on RpoE (RSP_1092) activity

**DOI:** 10.1186/s12866-018-1161-4

**Published:** 2018-02-27

**Authors:** Qingfeng Li, Tao Peng, Gabriele Klug

**Affiliations:** 1 Institut für Mikrobiologie und Molekularbiologie, Interdiziplinäres Forschungszentrum, Justus-Liebig-Universität Giessen, Heinrich-Buff-Ring 26-32, 35392 Giessen, Germany; 2 Present address: Department of Biology, Shantou University, Shantou, Guangdong China

**Keywords:** Alphaproteobacteria, *Rhodobacter sphaeroides*, General stress response, Oxidative stress, Membrane stress, PhyR, Alternative sigma factors, Anti sigma factor

## Abstract

**Background:**

A major role of the PhyR-NepR-σ(EcfG) cascade in the general stress response was demonstrated for some bacterial species and considered as conserved in Alphaproteobacteria. The σ(EcfG) factor activates its target genes in response to diverse stresses and NepR represents its anti-sigma factor. PhyR comprises a response regulator domain and a sigma factor domain and acts as anti-sigma factor antagonist. The facultative phototrophic alphaproteobacterium *Rhodobacter sphaeroides* harbours a PhyR homolog in the same genomic context as found in other members of this class.

**Results:**

Our study reveals increased expression of the *phyR* gene in response to superoxide, singlet oxygen, and diamide and also an effect of PhyR on *rpoE* expression. RpoE has a central role in mounting the response to singlet oxygen in *R. sphaeroides.* Despite these findings a mutant lacking PhyR was not significantly impeded in resistance to oxidative stress, heat stress or osmotic stress. However a role of PhyR in membrane stress is demonstrated.

**Conclusion:**

These results support the view that the effect of the PhyR-NepR-σ(EcfG) cascade on diverse stress responses varies among members of the Alphaproteobacteria. In the facultative phototroph *Rhodobacter sphaeroides* PhyR plays no major role in the general stress or the oxidative stress response but rather has a more specialized role in defense of membrane stress.

**Electronic supplementary material:**

The online version of this article (10.1186/s12866-018-1161-4) contains supplementary material, which is available to authorized users.

## Background

In their natural environment bacteria are exposed to changing conditions, which often cause stress. To survive these conditions they have evolved defense systems that allow adaptation to the changing environment. Many previous studies revealed a big overlap of the responses to a variety of different stress factors, considered as general stress response.

The PhyR-NepR-σ (EcfG) cascade was recognized as a core pathway regulating the general stress response in Alphaproteobacteria [1]. PhyR (phyllosphere regulator) was identified as a response regulator essential for plant colonization by *Methylobacterium extorquens* and promotes resistance to various stresses by controlling stress-related genes [2, 3]. In several Alphaproteobacteria, the *phyR* gene is in close proximity to the *nepR* and *ecfG* genes [1]. The σ(EcfG)-orthologous sigma factors RpoE2 (*Sinorhizobium meliloti*) and SigT (*Caulobacter crescentus*) induce large regulons in response to various stresses [4, 5]. In *M. extorquens,* NepR (negative regulator of the PhyR response) and PhyR control the activity of the σ(EcfG) sigma factor by acting as anti-sigma factor and as anti-sigma factor antagonist, respectively [6]. In the current model [1] NepR sequesters σ(EcfG) under non-stress conditions. Under stress conditions, the response regulator PhyR becomes phosphorylated, interacts with NepR and consequently releases σ(EcfG), which in turn associates with RNA polymerase.

*Rhodobacter sphaeroides* is a facultative phototrophic Alphaproteobacterium, which has been intensively studied in regard to its oxidative stress response, including singlet oxygen stress [7–9]. In photosynthetic bacteria (bacterio-) chlorophylls act as photosensitizers, which generate this harmful oxygen species when light and oxygen are present. The ECF sigma factor RpoE (RSP_1092) plays an important role in the singlet oxygen response of *R. sphaeroides.* Many genes are activated by RpoE [10, 11], however only a small subset directly [11]. Among the genes activated by RpoE is the alternative sigma factor RpoHII, which directly activates a much larger set of genes [12]. The RpoHII regulon has an overlap with the RpoHI regulon [13, 14], both sigma factors also have an important role in the general stress response [15]. Under non-stress conditions, RpoE is sequestered by the anti-sigma factor ChrR [16, 17], which is degraded upon singlet oxygen stress by the proteases DegS and RseP [18]. The RSP_2681 protein was also annotated as RpoE, but shares only 37% similarity with RSP_1092. No role of RSP_2681 in stress responses was reported to date. ChrR homologs, are also present in some Alphaproteobacteria besides *Rhodobacter* but lacking in others. While many Alphaproteobacteria harbour the PhyR-NepR-σ (EcfG) cascade as well as an RpoE/ChrR system, others like *Methylobacterium extorquens* or *Brucella melitensis* harbour the PhyR-NepR-σ (EcfG) cascade but lack a ChrR homolog.

A single PhyR homolog, RSP_1274, is also encoded in the *R. sphaeroides* chromosome and considering its function in other Alphaproteobacteria we hypothesized a function in the oxidative stress response and / or heat shock response in this organism. Our results do not support an important function of PhyR in theses stress responses for this phototrophic bacterium, however we observed an effect on membrane stress.

## Methods

### Bacterial strains and growth conditions

All strains, plasmids, and oligonucleotides used in this study are listed in Additional file 1. *Rhodobacter sphaeroides* was cultivated at 32 °C in minimal malate salt medium [19] in 50 ml Erlenmeyer flasks or flat bottles. Aerobic growth conditions with 160 to 180 μM dissolved oxygen were established by continuous shaking of 50 ml Erlenmeyer baffled flasks with 20 ml medium at 140 rpm or by gassing air into cultures in flat bottles. For microaerobic growth conditions, Erlenmeyer flasks with a culture volume of 80% were agitated at 140 rpm, resulting in a constant dissolved oxygen concentration of 25 to 30 μM during the mid exponential growth phase. *Escherichia coli* strains were grown in LB medium at 37 °C with shaking at 180 rpm or on solid growth medium, which contained 1.6% (*w*/*v*) agar. As necessary, antibiotics were added into liquid or solid medium at the following concentration: kanamycin (25 μg ml^− 1^); spectinomycin (10 μg ml^− 1^); tetracycline (1.5 μg ml^− 1^) (for *R. sphaeroides*); trimethoprim (50 μg ml^− 1^). Antibiotics were omitted from cultures and agar plates used for *R. sphaeroides* during stress experiments and zone inhibition assay.

### Construction of a *R. sphaeroides* PhyR (RSP_1274) deletion mutant

*Rhodobacter sphaeroides* strain ΔPhyR was generated by transferring the suicide plasmid pPHUΔRSP_1274:Sp into *R. sphaeroides* 2.4.1, and screening for insertion of the spectinomycin resistance cassette into the chromosome by homologous recombination. The suicide plasmid was also transferred to the strains ΔChrR and TF18 (lacks RpoE and ChrR) to create the respective double and triple mutant. Parts of the RSP_1274 gene of *R. sphaeroides* 2.4.1, together with upstream and downstream sequences were amplified by polymerase chain reaction (PCR) using oligonucleotides 1274_for1_KpnI/1274_rev2_EcoRI and 1274_for3_EcoRI/1274_rev4_XbaI (Additional file 1: Table S2). The amplified PCR fragments were cloned into the XbaI-EcoRI and EcoRI-KpnI sites of suicide plasmid pPHU281 [20], generating plasmid pPHUΔRSP_1274. A 2.2 kb fragment containing the spectinomycin cassette from pHP45Ω [21] was inserted into the EcoRI site of pPHUΔRSP_1274 to generate pPHUΔRSP_1274:Sp. This plasmid was transferred into *E. coli* strain S17-I and biparentally conjugated into *R. sphaeroides* 2.4.1 wild-type strain. Conjugants were selected on malate minimal medium agar plates containing spectinomycin (10 μg ml^− 1^) and subsequently tested for tetracycline sensitivity.

### RNA extraction, northern blot analysis, and RNAseq

For Northern blot analysis, samples from stress experiment were collected before (0 min) and 7 min after organic peroxide (360 μM of t-Butyl hydroperoxide (t-BOOH)) stress condition. Total RNA was isolated by the hot phenol method [22]. A total of 10 μg RNA was loaded per lane and separated on 10% polyacrylamide gels containing 7 M urea. RNA was transferred onto Biodyne B 0.45-μm membranes (Pall) by semidry electroblotting. For detection of Pos19 (photo-oxidative stress induced sRNA 19), the end-labeled oligonucleotide p-0019 was used. Membranes were exposed on phospho imaging screens (Bio-Rad) and analyzed with the Quantity One software (Bio-Rad).

RNAseq and dRNAseq are described in Remes et al. [23]. The RNA-seq data are available at the NCBI Gene Expression Omnibus database under accession number GSE71844.

### Quantitative real-time RT-PCR

The one step Brilliant III Ultra-Fast SYBR® QRT-PCR Master Mix Kit (Agilent) was used for reverse transcription followed by PCR as described in the manufacturer’s manual. For real-time RT-PCR, a final concentration of 4 ng μl^− 1^ of total RNA was run in a C1000 Thermal cycler (Bio-Rad) for relative quantification of mRNAs in each of the three independent experiments. Relative expression of target genes was calculated relative to expression of untreated samples and normalized to the housekeeping gene *rpoZ* according to Pfaffl [24].

### β-galactosidase assay

β-galactosidase assays were performed according to the method of Miller [25, 26]. At least three biological repeats were measured. In brief, cultures were inoculated from a single colony into 40 ml minimal salt medium and grown under microaerobic growth condition. Cultures were diluted to an OD_660_ of 0.2 in a flat bottle and allowed to double once under aerobic growth conditions in darkness. β-galactosidase activity was measured before (0 h), 1 and 3 h under singlet oxygen (high light 880 W m^− 2^ and 50 nM methylene blue) and organic peroxide (360 μM of t-Butyl hydroperoxide (t-BOOH)) stress.

### Zone of inhibition assay

All strains were grown in minimal salt medium to OD_660_ of 0.5. 500 μl of culture were mixed with 5 ml pre-warmed soft agar and poured on solid minimal salt medium. Disks were placed at the center of plates, 5 μl of 10 mM methylene blue, 750 mM t-BOOH, 700 mM diamide or 700 mM paraquat were added on the filter disks. The plates of methylene blue were incubated in the light (60 W lamp), other plates were incubated in the dark. The diameters of growth inhibition areas were measured after incubation at 32 °C for 3 days [18].

### Ultraviolet assay

All strains were cultured to an OD_660_ of 0.7–0.8 and diluted to a final dilution of 0.5 × 10^− 6^. 50 μl of the dilution was distributed on four plates. Plates were exposed to Ultraviolet (UV) light of 100 J m^− 2^ (254 nm) and incubated under the indicated temperature in the light or in the dark. The plates incubated in the light overnight were then transferred to the dark. Survival rates of UV exposed cells compared with non-exposed cells were calculated after incubating 3 days in the dark.

### Survival assays

All strains were cultured to an OD_660_ of 0.5 in microaerobic condition. For spot survival assays, ethanol (12%), SDS (0.015%) and EDTA (30 mM), polymyxin B (2.5 μg/ml) or CCCP (25 μM) were added. Growth of viable cells was monitored by spotting 5 μl from consecutive 10-fold dilutions onto agar plates after 30 and 60 min of ethanol, 15 and 30 min of SDS and EDTA. For counting survival cell numbers, cultures treated in the same way or treated for 60 min with polymyxin B (2.5 μg/ml), or 60 min of CCCP(25 μM) were further dilutes 10^− 1^ to 10^− 6^.(dependent on the strain) and 50 μl were spread on the plate. Colonies were counted after three days incubation at 32 °C in the dark.

## Results

### Genomic context of the *phyR* gene RSP_1274

**Fig. 1:**
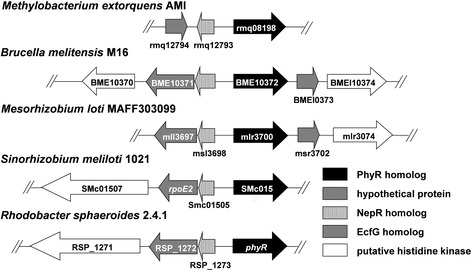
Genetic organization of the *phyR* locus in different Alphaproteobacteria

PhyR consisting of an amino terminal effector domain and a carboxy-terminal domain, was described first in *Methylobacterium extorquens* AM1 as a protein that regulates genes expression involved in general stress response. The amino terminal effector domain is a conserved general stress regulator in Alphaproteobacteria orthologous to sigma factors SigT of *Caulobacter crescentus* [4] and RpoE2 of *Sinorhizobium meliloti* [27]. The carboxy-terminal domain is also conserved. Phosphorylation of an aspartate in this domain leads to a conformational change which subsequently activates the effector domain [28]. Gene RSP_1274 of *R. sphaeroides* encodes a protein that shares 50%, 49%, 49% and 48% identity with the PhyR proteins from *Sinorhizobium meliloti*, *Methylobacterium extorquens* AM1, *Bradyrhizobium japonicum* and *Caulobacter crescentus*, respectively. As found for several other Alphaproteobacteria [1], an RNA polymerase sigma factor (RSP_1272) and a sensor histidine kinase (RSP_1271) are encoded upstream of the *phyR* gene (RSP_1274) on the opposite strand (Fig. 1). The RSP_1272 gene product shares 45% identity with RpoE2 from *S. meliloti* and 42% identity with SigT from *C. crescentus*. Other bacteria harbour the gene for the 61 aa NepR protein between the *ecfG* gene and the *phyR* gene. In this position *R. sphaeroides* encodes a 68 aa protein with 25% identity to NepR. Downstream of *phyR* a protein of the Crp-Fnr family is encoded. The gene arrangement for *R. sphaeroides* and selected Alphaproteobacteria is shown in Fig. 1.

Differential RNA sequencing (dRNAseq) revealed the transcriptional organization for these genes. This method compares RNA samples, which were treated with terminal exonuclease with untreated samples and thus discriminates between RNA 5′ ends with triphosphate (TSS, transcriptional start sites) and RNA with monophosphate at the 5′ end (processing sites) [29]. The data strongly suggest that RSP_1273 and RSP_1272 are transcribed together from a promoter which is located upstream of RSP_1273, opposite to the coding region of *phyR* (Additional file 2). Another TSS seems to be present for RSP_1271, however slightly downstream of the ATG start codon of the annotated protein. This ATG overlaps with the TAG terminator codon of RSP_1272 suggesting translational coupling.

### PhyR affects the resistance of *R. sphaeroides* to membrane stress but not to oxidative stress

In order to test the function of the PhyR homolog in *R. sphaeroides* stress response, we constructed a deletion strain which lacks the *phyR* gene and has a spectinomycin cassette inserted instead the doubling time of strain. ΔPhyR did not differ from the doubling time of the parental wild type 2.4.1 in aerobic conditions or during exposure to singlet oxygen (data not shown). There was also no difference in doubling time during osmotic stress (200 mM NaCl) in aerobic growth condition. Wild type and mutant strain also showed identical absorption spectra excluding an effect of PhyR on photosynthesis gene expression (data not shown).

**Fig. 2:**
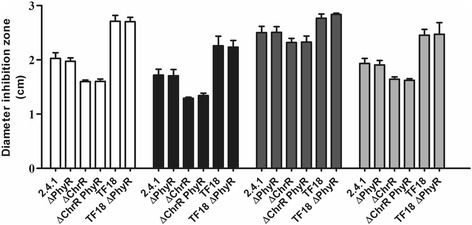
Inhibition of growth of the *R. sphaeroides* wild type strain 2.4.1 and of mutants lacking the *phyR* or *chrR* gene or *chrR* and *rpoE* (strain TF18) or *phyR* together with *chrR* or *rpoE/chrR*. Following agents were used in zone inhibition assays: t-BOOH (white bars), methylene blue (black bars), diamide (dark grey bars), or paraquat (light grey bars). Error bars indicate the standard deviation of zones of inhibition from three biological replicates. According to Students t-test none of the changes between PhyR deletion strains and the parental strains is significant (*P* ≤ 0.05)

Zone inhibition assays revealed no difference in resistance to singlet oxygen, t-BOOH, superoxide, or diamide between wild type and mutant (Fig. 2). Reaction of singlet oxygen with proteins, lipids and photopigments results in both direct damage and the formation of long-lived reactive organic peroxides. t-BOOH is used as model organic peroxide in our assays. In the same experiments we observed significantly increased resistance of a mutant lacking ChrR (RSP_1093) and decreased resistance in a mutant lacking RpoE (RSP_1092) and ChrR (strain TF18) against these stress factors (Fig. 2). Strains lacking ChrR and PhyR together or lacking RpoE/ChrR/PhyR showed the same stress resistance as the parental strains harbouring PhyR (Fig. 2). Thus an additional lack of PhyR did not alter the effects of higher RpoE activity (strain ΔChrR) or a lack of RpoE (strain TF18).

Since PhyR-dependent signaling also contributes to the heat stress response in other bacteria [6], we tested the effect of the deletion on growth at elevated temperature. Plates were incubated at 42 °C for 24 h after streaking and further incubation was at 32 °C for 72 h. These plates were compared to plates permanently grown at 32 °C. As previously described, we observed a clear growth defect of strains lacking RpoHI or RpoHI and RpoHII after heat stress [13]. Both sigma factors are known to be involved in the heat shock response of *R. sphaeroides* [12, 14]. There was no significant effect of elevated temperature on growth of the strain lacking PhyR (Additional file 3).

**Fig. 3:**
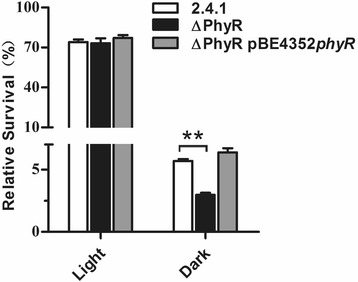
Survival of *R. sphaeroides* after UV light exposure. Cells of the indicated strains were exposed to UV light of 100 J m^− 2^ (254 nm), spread on agar plates and kept in the light (60 W lamp) or in the dark. The survival rates are given as the mean of three experiments with standard deviation. ** indicates a highly significant change (*P* ≤ 0.01) according to Students t-test

When *R. sphaeroides* cells were exposed to UV light and kept in the dark, the survival rate of the mutant strain was significantly lower than that of the wild type. When the cells were incubated in the light allowing photoreactivation, no difference in survival rates was observed for the two strains (Fig. 3). This suggests that PhyR does not affect the photolyase activity of *R. sphaeroides* [30, 31], but rather processes for UV repair that are light-independent.

Additional experiments addressed the resistance of *R. sphaeroides* wild type and mutant to membrane stress. Survival was monitored by spot plating assays following treatment with SDS and EDTA or treatment with ethanol. After 15 min of treatment with 0.015% SDS and 30 mM EDTA survival of the wild type was much better than that of the mutant (Additional file 4A) indicating a role of the PhyR homolog in defense of membrane stress. 30 min or 60 min after treatment with ethanol the wild type showed slightly better survival than the ΔPhyR mutant (Additional file 4B). When the PhyR mutant was complemented by a plasmid-encoded *phyR* gene (strain 2.4.1ΔPhyR (pBE::*phyR* eCFP)), survival was identical as for the wild type for both stresses (Additional file 4A and B).

**Fig. 4:**
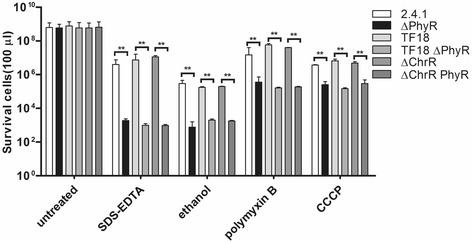
Survival rates of *R. sphaeroides* strains as determined by spread plating assays and colony counting before and after 15 min of addition of SDS (0,015%) and EDTA (30 mM), 30 min of ethanol (12%), 60 min of polymyxin (2.5 mg/ml), or 60 min of CCCP (25 μM). Error bars indicate the standard deviation from three biological replicates. ** indicates a highly significant change (*P* ≤ 0.01) according to Students t-test

We complemented these experiments by spread plating assays which confirmed a significant effect of PhyR on the survival in presence of SDS/EDTA or ethanol (Fig. 4c). We also included treatment with polymyxin that alters membrane permeability and the uncoupling reagent CCCP (carbonyl cyanide *m*-chlorophenyl hydrazone). The effect of the *phyR* mutation for these reagents was less pronounced than for SDS/EDTA or ethanol, nevertheless the survival rate in the mutant was significantly lower than in the wild type strain (Fig. 4).

To test whether the effect of PhyR on membrane stress is mediated via the RpoE/ChrR system we also constructed mutants lacking ChrR or ChrR/RpoE together with PhyR. The results clearly demonstrate that the effect on membrane stress is solely due to PhyR. Strains ΔChrR and TF18 showed the same resistance to membrane stress as the wild type (Fig. 4).

### Effect of *R. sphaeroides* PhyR on RpoE-dependent gene activation

**Fig. 5:**
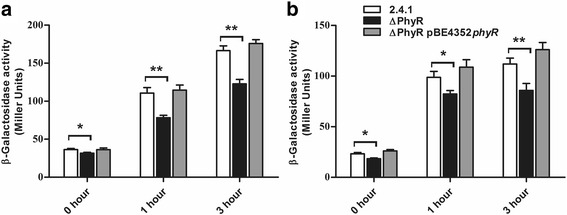
β-galactosidase actvity of *R. sphaeroides* strains harboring the reporter plasmid pPHU*phrAlacZ*. Cells were grown in aerobic conditions in the dark and were exposed to high light intensity (880 W m^− 2^) and 50 nM methylene blue (**a**) or to 360 μM of t-BOOH (**b**) for the indicated time periods. The mean of three experiments and standard deviations are shown. * indicates a significant change (*P* ≤ 0.05), ** indicates a highly significant change (*P* ≤ 0.01) according to Students t-test

RpoE (RSP_1092) of *R. sphaeroides* has an important function in the singlet oxygen stress response and also activates RpoHII in the general stress response. To elucidate a possible effect of PhyR on the activity of this sigma factor, we determined the β-galactosidase activity of a *phrA-lacZ* reporter plasmid. The *phrA* gene encodes a photolyase and is under direct control of RpoE. In strain TF18, which lacks RpoE and ChrR the β-galactosidase levels are very low in absence or presence of singlet oxygen (generated by addition of methylene blue and illumination) [30]. The ΔPhyR deletion strain showed an increase in β-galactosidase activity after 1 h and 3 h of singlet oxygen stress, which was however less than observed for the wild type (Fig. 5a). As a consequence the wild type showed significantly higher activity after 1 h and 3 h of stress (Fig. 5a). *phrA-lacZ* activity was also monitored after treatment with t-BOOH (Fig. 5b). The β-galactosidase activity was significantly lower in the mutant after 1 h and 3 h of stress compared to the wild type. When the *phyR* gene was introduced into the strain that has *phyR* deleted from the chromosome, we observed ß-galactosidase activity similar or higher to that of the wild type, proving that altered RpoE activity in the mutant strain is indeed a consequence of the lack of PhyR.

Another gene, which is under direct control of RpoE encodes the small RNA Pos19 [32, 33]. Northern Blot analysis revealed a stronger induction of Pos19 in the wild type compared to the *phyR* mutant upon treatment with organic peroxide (Additional file 5).

### Effect of *R. sphaeroides* PhyR on stress-dependent mRNA levels

**Fig. 6:**
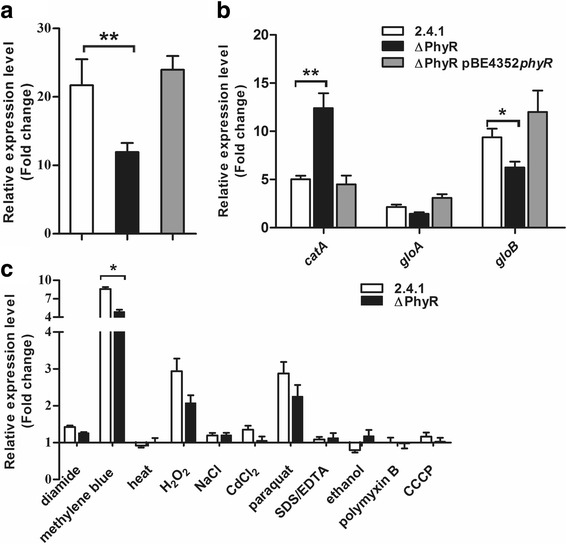
**a** Levels of *rpoE* mRNA as determined by real time RT PCR in wild type 2.4.1 (white bars), *phyR* mutant (black bars) and complemented strain (grey bars). The fold change of 7 min versus 0 min under t-BOOH after normalization to *rpoZ* mRNA level is shown. **b** Levels of relative expression are shown for *catA* in response to hydrogen peroxide and *gloA*, *gloB* in response to ^1^O_2_ exposure in wild type 2.4.1 (white bars), *phyR* mutant (black bars) and complemented strain (grey bars). Exposure to hydrogen peroxide or ^1^O_2_ was performed for 7 min. Values for relative expression levels represent the increase in gene expression compared to that of the control at time point 0 min and were normalized to mRNA levels determined for *rpoZ*. The mean of three experiments with standard deviation is shown. **c** Relative *rpoE* mRNA levels under different stress conditions as determined by real time RT PCR in the wild type and the mutant lacking PhyR. * indicate a significant change (*P* ≤ 0.05), ** indicate a highly significant change (*P* ≤ 0.01) according to Students t-test

Our data strongly indicate reduced RpoE-dependent gene activation in the strain lacking PhyR. Real time RT-PCR analyses revealed that the *rpoE* mRNA level after treatment with t-BOOH increases significantly stronger in the wild type and in the complemented mutant than in ΔPhyR (Fig. 6a). We also tested the effect of PhyR on some other genes with a role in the oxidative stress response in *R. sphaeroides* [34, 35]. After 7 min of hydrogen peroxide the *catA* (RSP_2779*)* mRNA level for catalase increased significantly more in the PhyR mutant than in the wild type (Fig. 6c). For the mRNAs *gloA* (RSP_0392) and *gloB* (RSP_2294) for putative glyoxalases, higher induction in response to singlet oxygen was however observed for the wild type and the complemented PhyR mutant compared to the PhyR mutant(Fig. 6b). The *gloA* and *gloB* genes are preceded by RpoHII-dependent promoters (14), while *catA* is not preceded by a promoter sequence for an alternative sigma factor. Since the *rpoHII* gene is under control of RpoE, changed expression levels of *gloA* and *gloB* mRNAs are most likely a consequence of the PhyR effect on RpoE activity.

**Fig. 7:**
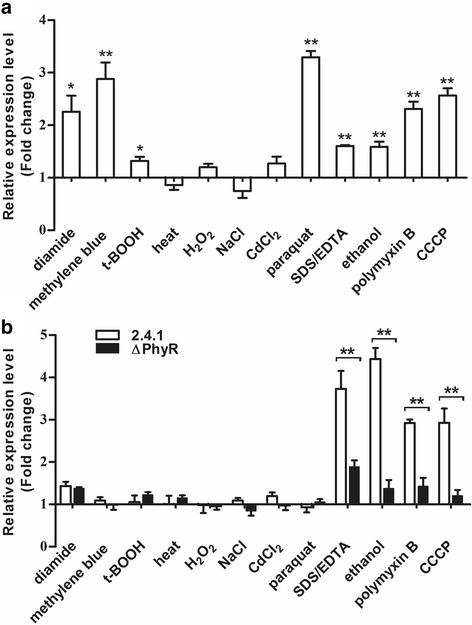
Levels of *phyR* (**a**) and RSP_1272 (**b**) mRNAs under various stresses as determined by real time RT PCR. The following reagents were added to aerobic cultures at OD_660_ of 0.4 and samples were collected immediately before (0 min) and 7 min after addition: 0.2 μM methylene blue and high light (880 W m^− 2^), 360 μM t-BOOH, 1 mM H_2_O_2_, 250 μM paraquat, 500 μM diamide, 500 mM NaCl, or 10 μM CdCl_2_. For heat shock, microaerobic cultures were shifted to 42 °C at time 0 min or the following reagents were added: 0.005% SDS and 1 mM EDTA, 2.5% ethanol, 1 μg/ml polymyxin B or 10 μM CCCP, and samples were collected at 0 min and 7 min. The mean of three experiments with standard deviation is shown. * indicate a significant change (*P* ≤ 0.05), ** indicate a highly significant change (*P* ≤ 0.01) according to Students t-test

We also followed *rpoE* mRNA levels in response to other stress conditions in the wild type and the ΔPhyR mutant strain (Fig. 6c). A significant difference in *rpoE* mRNA level between the two strains was observed for singlet oxygen stress (methylene blue in the light). Diamide, heat, SDS/EDTA or ethanol had only minor effects on *rpoE* mRNA levels, while superoxide (paraquat treatment) and hydrogen peroxide resulted in increased *rpoE* mRNA levels, which were slightly lower in the mutant, but these differences were statistically not significant (Fig. 6b). We also tested how diverse stresses affect *phyR* and RSP_1272 (−σ (EcfG)) mRNA levels by real time RT PCR (Fig. 7). While singlet oxygen (methylene blue), superoxide (paraquat) and diamide resulted in 2–3 fold increase of the *phyR* mRNA level, none of the other stresses including membrane stress led to marked changes in *phyR* mRNA levels. Membrane stress was however the only stress factor that resulted in increased RSP_1272 mRNA levels and this increase is clearly dependent on PhyR.

## Discussion

An important role for the PhyR-NepR-σ (EcfG) cascade in regulating the general stress response was observed in several members of the Alphaproteobacteria [1]. Genes encoding proteins with good homology to the proteins of the PhyR-NepR-σ (EcfG) cascade are also present in *R. sphaeroides* and are found in a similar chromosomal arrangement (Fig. 1). A similar role of PhyR in stress responses of *R. sphaeroides* as in other Alphaproteobacteria was conceivable. A *R. sphaeroides* strain lacking PhyR showed similar response as the parental wild type to singlet oxygen, hydrogen peroxide, superoxide, organic peroxides, diamide, heat or salt stress. We conclude that other proteins like the alternative sigma factors RpoE, RpoHI, and RpoHII are indeed the main regulators of the general stress response in *R. sphaeroides*.

Our data demonstrate however that expression of the *phyR* gene is modulated in response to some stresses and we found significant differences between wild type and mutant in response to membrane and UV stress. This suggests that PhyR has a more specialized role in *R. sphaeroides*. Up to now, the role of PhyR in stress responses was not analyzed for any member of the Rhodobacterales. Therefore it is possible that a more specialized function of PhyR is not limited to *R. sphaeroides* but may apply to a certain sub-branch of the Alphaproteobacteria.

While the *phyR* mRNA levels did not respond to membrane stress, the levels of the RSP_1272 mRNA encoding the σ(EcfG) protein of the PhyR-NepR-σ (EcfG) locus, was clearly increased in response to membrane stress, but not by other stress factors. This supports the view that the PhyR-NepR-σ (EcfG) cascade in *R. sphaeroides* has a main role in the defense of membrane stress. Increased RSP_1272 mRNA levels were dependent on PhyR in agreement with signal transfer within the PhyR-NepR-σ (EcfG) cascade.

Furthermore our data provide clear evidence for an effect of PhyR on *rpoE* mRNA level and RpoE-dependent gene activation (Figs. 5, 6 and Additional file 5). Why is the influence of PhyR on RpoE activity not manifested as altered stress resistance in the mutant? In mutants lacking ChrR or RpoE and ChrR together (strain TF18) the change in *rpoE* mRNA level is of course much more pronounced than in the PhyR mutant under the tested conditions. ΔChrR or TF18 mutants are clearly affected in stress resistance, but additional mutation of *phyR* did not increase this effect implying that PhyR can not even partially compensate the loss of these main regulators. Many investigations in the past have demonstrated that complex regulatory networks including protein and RNA regulators are involved in controlling and balancing stress responses [36–38]. The moderate changes of RpoE activity caused by the lack of PhyR may not be sufficient to cause a clear phenotype since compensation by other players in the regulatory network may take place. In *R. sphaeroides* the PhyR-NepR-σ(EcfG) cascade may also have a function in balancing some stress responses rather than triggering such responses. A cross talk of the PhyR-NepR-σ(EcfG) cascade to the RpoE/ChrR system has not been reported for other Alphaproteobacteria to date.

## Conclusions

Our results demonstrate that PhyR has no major role in the general stress response of the Alphaproteobacterium *R. sphaeroides* as reported before for other bacterial species [1, 39, 40]. We could attribute a role of PhyR in defense of membrane stress and survival of UV light in the dark in *R. sphaeroides*, supporting a more specialized function in this bacterium. PhyR has no major contribution to the complex regulatory network (e.g.: 8, 9, 15, 29, 30) of protein and sRNA regulators that control the oxidative stress response in *R. sphaeroides*.

## Additional files

Additional file 1: Strains and plasmids (**Table S1**), Oligodeoxynucleotides (**Table S2**) used in this study. (PDF 163 kb)

Additional file 2: Schematic representation and RNA-seq read coverage of the *phyR* operon in *Rhodobacter sphaeroides*. Blue: Read coverage of the *phyR* operon in *R. sphaeroides* visualized by the Integrated Genome Browser. Black: Genes are represented by black boxes. The direction of the arrow indicates the direction of transcription. (PDF 66 kb)

Additional file 3: Growth of the *Rhodobacter sphaeroides* wild type 2.4.1 and various mutant strains after heat shock. Cultures were grown at 32 °C to exponential phase in microaerobic conditions and diluted to OD_660_ of 0.1. For each strain 5 μl of diluted culture were spread on agar plates and incubated under the indicated temperature in the dark. The agar plates incubated at 42 °C were shifted to 32 °C after 24 h. (PDF 791 kb)

Additional file 4: Spot survival assays for *R. sphaeroides* and *phyR* mutant after membrane stress. All strains were cultured to an OD_660_ of 0.5 in microaerobic condition. 5 μl from consecutive 10-fold dilutions were spotted onto agar plates before and after 15 and 30 min of SDS (0.015%) and EDTA (30 mM) treatment(A) or after 30 and 60 min of ethanol (12%) treatment. (PDF 1323 kb)

Additional file 5: Northern blot analysis of Pos19. Cultures were treated with t-BOOH and samples taken at time point 0 and 7 min. Pos19 bands were normalized to the 5S rRNA and the calculated fold change is indicated. (PDF 669 kb)

## Abbreviations

CCCP: Carbonyl cyanide *m*-chlorophenyl hydrazone
EcfG: Extracytoplasmic function sigma factor
t-BOOH: t-Butyl hydroperoxide
TSS: Transcriptional start sites
UV: Ultraviolet light
β-Gal: β-galactosidase
