# Supplementary material for: The PhyR homolog RSP_1274 of Rhodobacter sphaeroides is involved in defense of membrane stress and has a moderate effect on RpoE (RSP_1092) activity
Source: BMC Microbiol. 2018 Feb 27;18:18. doi: 10.1186/s12866-018-1161-4 (PMC5830050; doi:10.1186/s12866-018-1161-4)
Supplement: Supplementary file 1 — Strains and plasmids (Table S1), Oligodeoxynucleotides (Table S2) used in this study. (PDF 163 kb) [file 12866_2018_1161_MOESM1_ESM.pdf]

Table 1 Strains and plasmids used in this study

Strain or plasmid	Description or relevant features	Source/reference
Strains		
E. coli		
S17-1	*recA pro hsdR RP4-2*-Tc::Mu Km::Tn7 tra^{+} Km^{r} Sp^{r}	[1]
JM109	*recA1 supE44 endA1 hsdR17 gyrA96 relA1 thi (lac-proAB)*	New England Biolabs
R. sphaeroides		
2.4.1	Wild type	
TF18	*rpoE chrR* mutation in 2.4.1, Tp^{r}	[2]
Δ$rpoH_{II}$	2.4.1 $rpoH_{II}$::Sp^{r} cassette	[3]
Δ$rpoH_{I}$	2.4.1 *rpoHI*::Km^{r} cassette	[4]
Δ$rpoH_{I}$Δ$rpoH_{II}$	2.4.1 Δ$rpoH_{II}$$rpoH_{I}$ ::Km^{r} cassette	[4]
ΔPhyR	2.4.1 *phyR*::Sp^{r} cassette	This study
ΔChrR	2.4.1 *chrR*::Tp^{r} cassette	[2]
TF18ΔPhyR	TF18 *phyR*::Sp^{r} cassette	This study
ΔChrR PhyR	ΔChrR *phyR*::Sp^{r} cassette	This study
Plasmids		
pPHU281	Suicide vector for *R. sphaeroides*. Tc^{r}	[5]
pPHUΔRSP_1274:: Sp	pPHU281 with Sp cassette, flanked by the upstream and downstream regions of RSP_1274, Tc^{r}	This study
pBE4352	Km^{r}, pBE containing RSP_4352 promoter	[6]
pBE *phyR*	Km^{r}, pBE4352 containing *phyR* fragment for *cfp* fusion	This study
pHP45Ω	Sp cassette, Sp^{r}	[7]
pJET1.2	Ap cassette, Ap^{r}	Thermo
pPHU*phrAlacZ*	pPHU234 with *phrA* upstream-region, Tc^{r}	[8]

Table 2. Oligodeoxynucleotides used in this study

Name	Sequence	Purpose
1274_for1_kpnI	5´-CGCAGGTTGGTACCTTCGGTG-3´	RSP_1274 deletion
1274_rev2_EcoRI	5´-CGGTGCCCGAATTCGGTTCCG-3´	RSP_1274 deletion
1274_for3_EcoRI	5´-GGAGCCGGAATTCCTCATCACC-3´	RSP_1274 deletion
1274_rev4_XbaI	5´-GAAGATTGGGCGTCTAGAAGGCGC-3´	RSP_1274 deletion
1274check-for	5´-CGAAGTTCGTCCAGGCCTTC-3´	RSP_1274 deletion
1274check-rev	5´-TCTTGCGCAGACGATCCTCG-3´	RSP_1274 deletion
1274check-up	5´-CAGGATGGTGAAGAGCCAGG-3´	RSP_1274 deletion
1274check-down	5´-AACATGTCACAGTTTAATGCGGG-3´	RSP_1274 deletion
1274CF	5´-CATATGATGACCTCCGACACGACG-3´	RSP_1274 cloning
1274CR	5´-GGATCCGGCGCTGAGCGTTTCGGTG-3´	RSP_1274 cloning
1274RTF	5´-GGGACCAACCTGCCGTAT-3´	RT-PCR for RSP_1274
1274RTR	5´-CCAGACGAGATGGAACGC-3´	RT-PCR for RSP_1274
RT1272F	5´-CCTGCGGAACACCTTCTA-3´	RT-PCR for RSP_1272
RT1272R	5´-ATCCTCATAGGCGAAGCC-3´	RT-PCR for RSP_1272
p-0019	5´-GAGATAGCTCATCGGTCAGGTCC-3´	Northern probe for Pos19
p-5S	5´-CTTGAGACGCAGTACCATTG-3´	Northern probe for 5S rRNA
RpoZ-A	5´-ATCGCGGAAGAGACCCAGAG-3´	RT-PCR for *rpoZ*
RpoZ-B	5´-GAGCAGCGCCATCTGATCCT-3´	RT-PCR for *rpoZ*
rpoE-A	5´-GTCTGGCAGAAGGCTCAT-3´	RT-PCR for *rpoE*
rpoE-B	5´-GTTCTCCTGCTGCATCTC-3´	RT-PCR for *rpoE*
CatE-A	5´-CTATCCGCTGATCGAGGT-3´	RT-PCR for *catE*
CatE-B	5´-GTCGGCATAGGAGAAGAC-3´	RT-PCR for *catE*
GloA-A	5´-GTCGAACTCACCTACAAC-3´	RT-PCR for g*loA*
GloA-B	5´-CGCACATGTCGTAGATATC-3´	RT-PCR for *gloA*
GloB-A	5´-GAACAATTACGCCTTCTC-3´	RT-PCR for *gloB*
GloB-B	5´-CATCAGCTGGTAGCTCTC-3´	RT-PCR for *gloB*

Supp References

1. Simon R, Oconnell M, Labes M, Puhler A. Plasmid Vectors for the Genetic-Analysis and Manipulation of Rhizobia and Other Gram-Negative Bacteria. Methods in Enzymology. 1986;118:640-59.
2. Schilke BA, Donohue TJ. ChrR positively regulates transcription of the *Rhodobacter sphaeroides* cytochrome c2 gene. Journal of bacteriology. 1995;177(8):1929-37.
3. Nuss AM, Glaeser J, Klug G. RpoH(II) activates oxidative-stress defense systems and is controlled by RpoE in the singlet oxygen-dependent response in Rhodobacter sphaeroides. J Bacteriol. 2009;191(1):220-30.
4. Nuss AM, Adnan F, Weber L, Berghoff BA, Glaeser J, Klug G. DegS and RseP Homologous Proteases Are Involved in Singlet Oxygen Dependent Activation of RpoE in *Rhodobacter sphaeroides*. Plos One. 2013;8(11).

5. Hubner P, Willison JC, Vignais PM, Bickle TA. Expression of Regulatory Nif Genes in Rhodobacter-Capsulatus. J Bacteriol. 1991;173(9):2993-9.

6. Remes B, Eisenhardt BD, Srinivasan V, Klug G. IscR of Rhodobacter sphaeroides functions as repressor of genes for iron-sulfur metabolism and represents a new type of iron-sulfur-binding protein. Microbiologyopen. 2015;4(5):790-802.

7. Prentki P, Krisch HM. In vitro insertional mutagenesis with a selectable DNA fragment. Gene. 1984;29(3):303-13.

8. Hendrischk AK, Braatsch S, Glaeser J, Klug G. The *phrA* gene of *Rhodobacter sphaeroides* encodes a photolyase and is regulated by singlet oxygen and peroxide in a sigma(E)-dependent manner. Microbiology. 2007;153(Pt 6):1842-51.
