# Supplementary figures and images for: The PhyR homolog RSP_1274 of Rhodobacter sphaeroides is involved in defense of membrane stress and has a moderate effect on RpoE (RSP_1092) activity
Source: BMC Microbiol. 2018 Feb 27;18:18. doi: 10.1186/s12866-018-1161-4 (PMC5830050; doi:10.1186/s12866-018-1161-4)

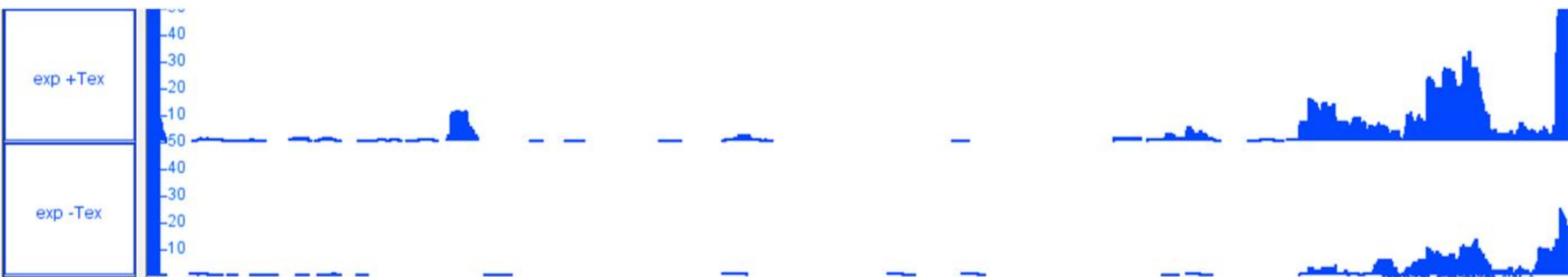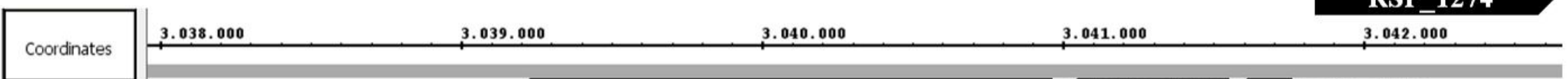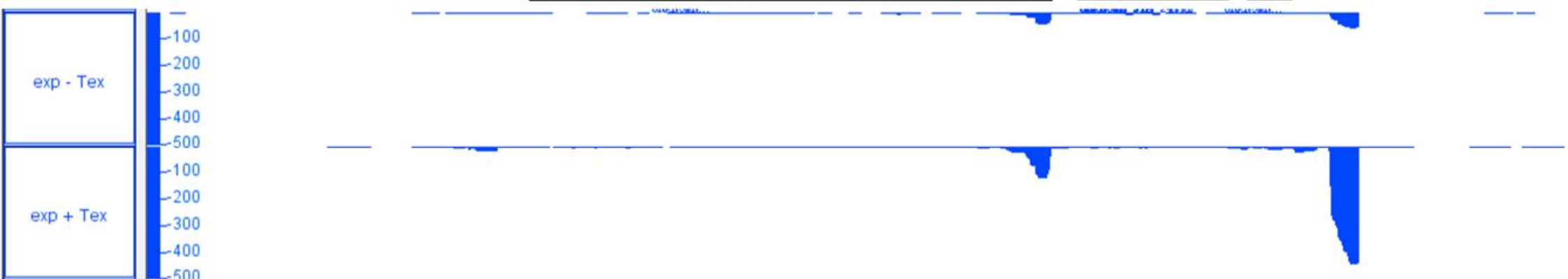

RSP\_1271 RSP\_1272 RSP\_1273

Supplement: Supplementary file 2 — Schematic representation and RNA-seq read coverage of the phyR operon in Rhodobacter sphaeroides. Blue: Read coverage of the phyR operon in R. sphaeroides visualized by the Integrated Genome Browser. Black: Genes are represented by black boxes. The direction of the arrow indicates the direction of transcription. (PDF 66 kb) [file 12866_2018_1161_MOESM2_ESM.pdf]

$\Delta\text{PhyR}$

$\Delta\text{rpoHI}$

2.4.1

$\Delta\text{rpoHII}$

TF18

$\Delta\text{rpoHI}\Delta\text{rpoHII}$

32°C 72 h

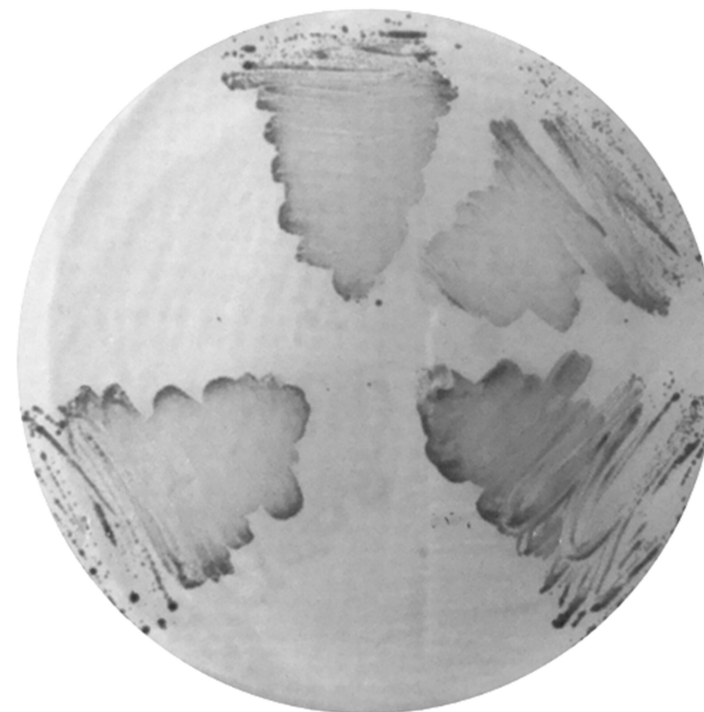

42°C 24 h

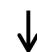

32°C 72 h

Supplement: Supplementary file 3 — Growth of the Rhodobacter sphaeroides wild type 2.4.1 and various mutant strains after heat shock. Cultures were grown at 32 °C to exponential phase in microaerobic conditions and diluted to OD660 of 0.1. For each strain 5 μl of diluted culture were spread on agar plates and incubated under the indicated temperature in the dark. The agar plates incubated at 42 °C were shifted to 32 °C after 24 h. (PDF 791 kb) [file 12866_2018_1161_MOESM3_ESM.pdf]

A

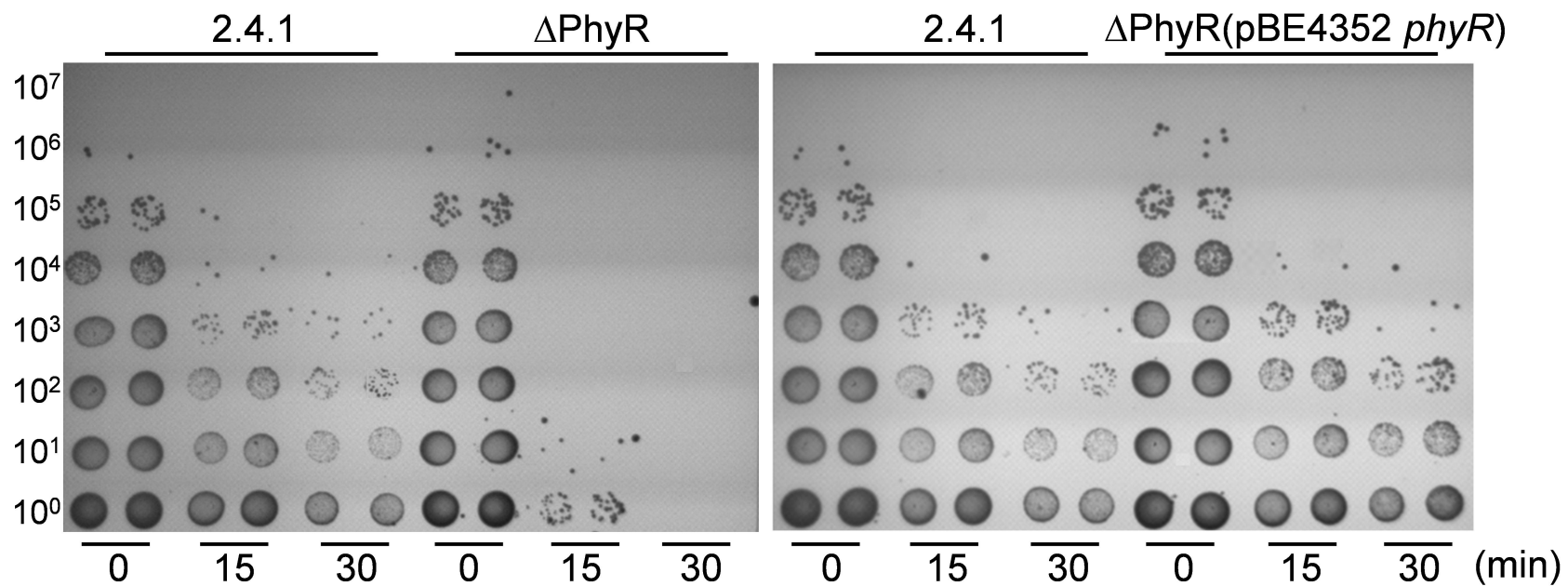

B

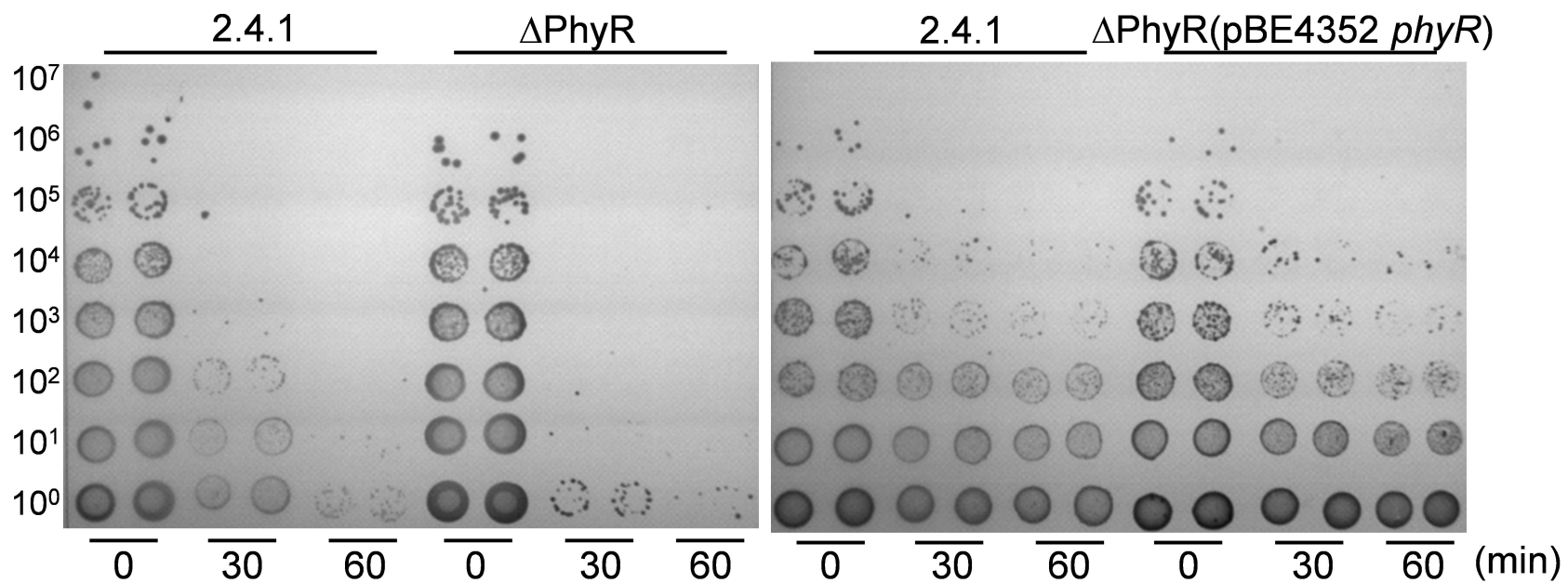

Supplement: Supplementary file 4 — Spot survival assays for R. sphaeroides and phyR mutant after membrane stress. All strains were cultured to an OD660 of 0.5 in microaerobic condition. 5 μl from consecutive 10-fold dilutions were spotted onto agar plates before and after 15 and 30 min of SDS (0.015%) and EDTA (30 mM) treatment(A) or after 30 and 60 min of ethanol (12%) treatment. (PDF 1323 kb) [file 12866_2018_1161_MOESM4_ESM.pdf]

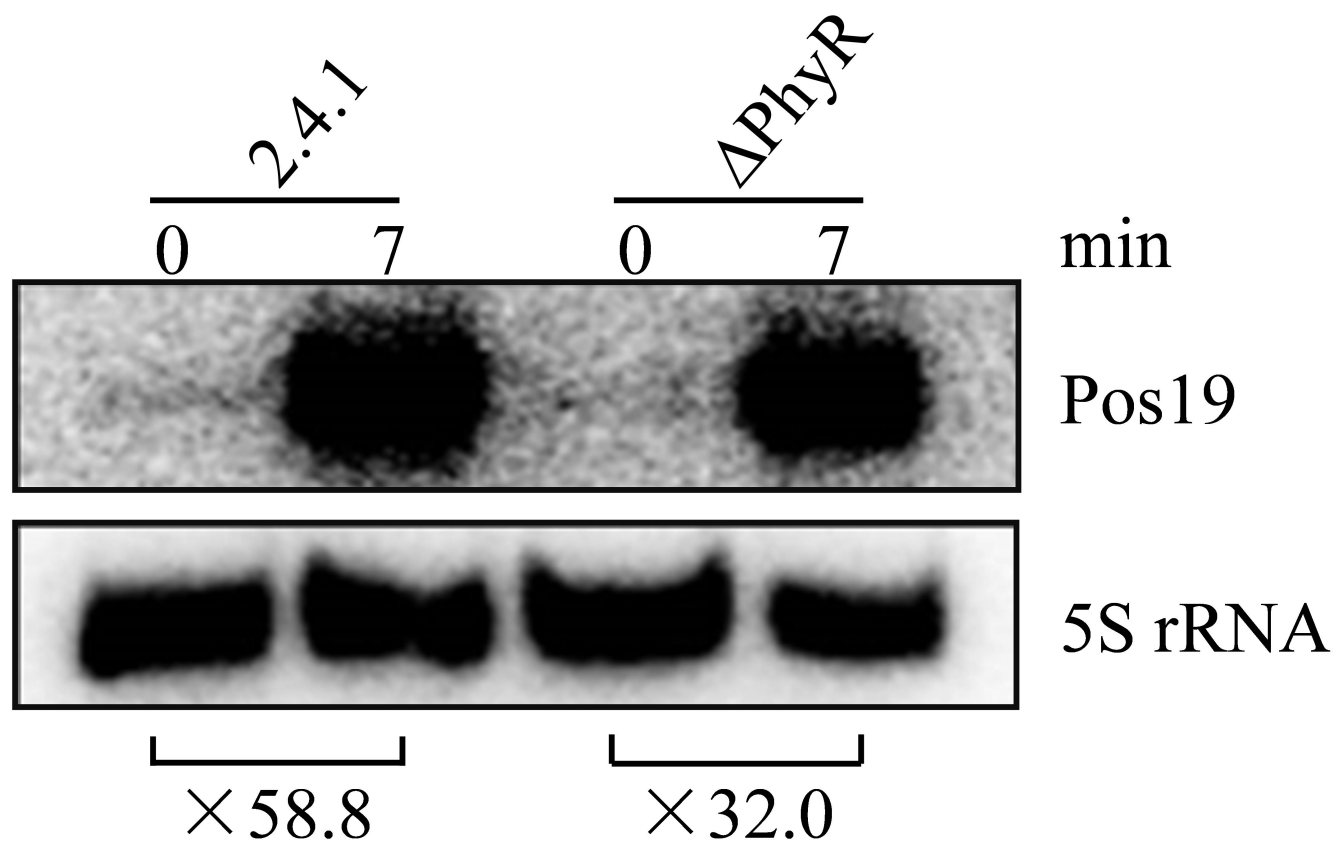

Supplement: Supplementary file 5 — Northern blot analysis of Pos19. Cultures were treated with t-BOOH and samples taken at time point 0 and 7 min. Pos19 bands were normalized to the 5S rRNA and the calculated fold change is indicated. (PDF 669 kb) [file 12866_2018_1161_MOESM5_ESM.pdf]
